# Diphenyl Disulfide Induces Nonapoptotic Paraptosis in Breast Cancer Cells via ROS-Mediated ER Stress

**DOI:** 10.7150/ijms.123945

**Published:** 2026-05-01

**Authors:** Sheng-Yuan Chen, Chang-Yi Wu, Wen-Hsiung Pan, Sheng-Kai Hsu, Wen-Tsan Chang, Yen-Chun Chen, Chen-Xi He, En-De Shu, Zhi-Hong Wen, Chien-Chih Chiu

**Affiliations:** 1 Department of Marine Biotechnology and Resources, National Sun Yat-sen University, Kaohsiung 804, Taiwan.; 2 Zuoying Armed Forces General Hospital, Kaohsiung 813, Taiwan.; 3 Department of Biological Sciences, National Sun Yat-sen University, Kaohsiung, 804, Taiwan.; 4 Department of Biotechnology, Kaohsiung Medical University, Kaohsiung 807, Taiwan.; 5 PhD Program in Life Sciences, College of Life Science, Kaohsiung Medical University, Kaohsiung 807, Taiwan.; 6 Division of General and Digestive Surgery, Department of Surgery, Kaohsiung Medical University Hospital, Kaohsiung 807, Taiwan.; 7 Department of Surgery, School of Medicine, College of Medicine, Kaohsiung Medical University, Kaohsiung 807, Taiwan.; 8 National Museum of Marine Biology & Aquarium, Pingtung, 944, Taiwan.; 9 Center for Cancer Research, Kaohsiung Medical University, Kaohsiung 807, Taiwan.; 10 Department of Medical Research, Kaohsiung Medical University Hospital, Kaohsiung 807, Taiwan.; 11 Drug Development and Value Creation Research Center, Kaohsiung Medical University, Kaohsiung 807, Taiwan.

**Keywords:** breast cancer, diphenyl disulfide, paraptosis, oxidative stress, ER stress, chemoresistance, nonapoptotic cell death

## Abstract

Breast cancer is the most common cancer in women and a leading cause of cancer-related mortality worldwide. Most anticancer drugs act against cancer cells by inducing apoptotic pathways; unfortunately, chemoresistance, especially apoptosis resistance, contributes to poor outcomes in patients with breast cancer. Paraptosis is a type of nonapoptotic programmed cell death characterized mainly by cytoplasmic vacuolization and endoplasmic reticulum (ER)/mitochondrial swelling, which may provide a promising strategy for overcoming chemotherapy resistance. We investigated the effects of diphenyl disulfide (DPDS) on two breast cancer cell subtypes, MDA-MB-231 (triple-negative) and MCF-7 (luminal A). Our findings demonstrate that DPDS exerts significant cytotoxic effects on these two breast cancer cell lines. In addition, DPDS induced the accumulation of vacuoles in cells and downregulated the paraptosis marker Alix while increasing the expression of the ER stress markers BIP/Grp78 and IRE-1 and oxidative stress owing to the accumulation of reactive oxygen species (ROS), eventually inducing paraptosis. In contrast, pretreatment with N-acetylcysteine (NAC) significantly restored cell survival and reduced BIP expression. Our study highlights the potential of DPDS to target two cell death pathways, representing a novel therapeutic strategy for patients with breast cancer resistant to apoptosis-inducing therapies

## Introduction

Breast cancer is the most common malignancy among women worldwide. The primary treatment modalities for cancer include surgical resection and chemotherapy. To date, the main chemotherapeutic strategy remains the induction of cancer cell apoptosis 1. However, in recent years, dysregulated apoptosis has been shown to contribute to drug resistance in patients with breast cancer, leading to poor patient prognosis and affecting overall survival rates 2-5. For example, the upregulation of survivin or X-linked inhibitor of apoptosis (XIAP) is associated with increased resistance to taxane and anthracycline 6. Furthermore, overexpression of the antiapoptotic protein BAG cochaperone 3 (BAG3) is observed in chemoresistant triple-negative breast cancer (TNBC) cell lines, while depletion of BAG3 potently restores chemosensitivity and is accompanied by reduced Bcl-2 and Bcl-xL levels 7.

Despite the acquired chemoresistance, especially apoptosis resistance, of breast cancer cells, various nonapoptotic programmed cell death pathways (e.g., ferroptosis, pyroptosis, and paraptosis) that may provide alternative strategies for overcoming chemoresistance mediated by antiapoptotic effects in breast cancer patients have been identified. For example, the induction of pyroptosis increases the chemosensitivity of MCF-7 cells to paclitaxel through the demethylation of gasdermin E (GSDME) 8. In addition, a previous study suggested that the inhibition of ferroptosis by the splicing factor SR-rich splicing factor 1 (SRSF1) confers resistance to cisplatin in TNBC cells 9. Recently, it was also demonstrated that a monacolin-K loaded MIL-100(Fe) metal-organic framework could effectively trigger ferroptosis in metastatic triple-negative breast cancer cells, highlighting the potential of utilizing innovative drug delivery systems to activate nonapoptotic pathways 10. These findings highlight that the initiation of nonapoptotic programmed cell death may reverse chemoresistance mediated by antiapoptotic activities. Among the various forms of nonapoptotic cell death, paraptosis is a novel form characterized by cytoplasmic vacuolization and ER/mitochondrial swelling 11. Moreover, the expression of the protein AIP-1/Alix, which is located within the cytoplasm and can induce apoptosis in cells, is negatively regulated during paraptosis 12-14.

Unlike apoptosis, which is often dysregulated in breast cancer cells because of the upregulation of antiapoptotic proteins, paraptosis involves an alternative cell death pathway that is less susceptible to these resistance mechanisms and presents a novel therapeutic avenue for breast cancer patients by bypassing the antiapoptotic mechanisms that contribute to chemoresistance 15, 16. Recently, the pyrazolo derivative YRL1091 was reported to trigger paraptosis, which serves as a promising therapeutic strategy to eliminate cancers that are resistant to conventional chemotherapy 17.

Reactive oxygen species (ROS) are oxygen-containing, chemically reactive molecules that are generated primarily within cells via mitochondria 18, 19. An appropriate level of ROS helps cells maintain the balance of intracellular redox reactions and facilitates normal cellular growth by participating in intracellular signaling 20. On the other hand, the ER is responsible for repairing misfolded proteins to prevent the secretion of incorrectly folded proteins, which could disrupt cellular physiology. Studies indicate that various cellular stresses, such as hypoxia 21 and excessive ROS accumulation 21, 22, can lead to protein misfolding in the ER that may result in ER stress 23.

Disulfide bond-based compounds have been reported to have anticancer activities. For example, bis[2-(acylamino)phenyl] disulfide, 1,2-bis(4-chlorophenyl) disulfide, 1,2-bis(4-methoxyphenyl) disulfide, and 1,2-bis(4-nitrophenyl) disulfide have been reported to have anticancer effects 24. Notably, bis[2-(acylamino)phenyl] disulfide triggered JNK-mediated apoptosis in human colon cancer cells 25. Furthermore, diallyl disulfide (DADS) inhibits the growth of and induces apoptosis in colorectal cancer (CRC) and breast cancer cells 26-28. Interestingly, our recent study demonstrated that DPDS, which has a similar structure to DADS, induces apoptosis through Bax cleavage in two breast cancer cell lines, MCF-7 and MDA-MB-231 29.

Although our recent study demonstrated that DPDS induces apoptosis in breast cancer cells 29, whether DPDS can also trigger nonapoptotic cell death pathways remains unclear. Paraptosis, a form of nonapoptotic programmed cell death characterized by cytoplasmic vacuolization and ER swelling, has emerged as an alternative strategy to overcome resistance to apoptosis in cancer cells 30-34. Given that paraptosis is closely associated with ER stress and oxidative stress and that the structure of DPDS contains a disulfide capable of inducing redox imbalance, we hypothesized that DPDS may induce paraptosis in breast cancer cells through ROS-mediated ER stress. In this study, we report that DPDS induces extensive cytoplasmic vacuolization and a reduction in the expression of the paraptosis inhibitor Alix while triggering significant ER stress and oxidative stress. Notably, our results revealed that the attenuation of ER stress partially restored cell viability, confirming the critical role of ER stress in DPDS-induced cell death.

## Materials and Methods

### Cell culture and maintenance

The human breast cancer cell lines MDA-MB-231 and MCF-7 were obtained from ATCC (Manassas, VA, USA) and Bioresource Collection and Research Center (BCRC, Hsin-Chu City, Taiwan), respectively. Both cell lines were maintained in DMEM/F12 (3:2 ratio) supplemented with 10% fetal bovine serum, 100 units/mL streptomycin/penicillin, and 2 mM glutamine at 37 °C in a humidified atmosphere with 5% CO_2_. DPDS was dissolved in DMSO as a stock solution; the final concentration of DMSO in all treatment groups was kept below 0.1% (v/v) to minimize vehicle toxicity.

### Colony formation assay

A total of 200 MDA-MB-231 or MCF-7 cells were seeded in a 12-well culture plate and incubated for 24 h at 37 °C with 5% CO_2_. The cells were subsequently treated with the indicated concentrations of DPDS (2, 5, or 10 μM) for 72 h, as described in our previous study 35. After treatment, the supernatant was collected, and fresh culture medium was added to continue cultivation for 10 days. The cells were washed once with 1× PBS and then fixed with 4% paraformaldehyde (PFA; #P6148; Sigma‒Aldrich) for 10 min. After fixation, the cells were washed three times with 1× PBS and stained with 1× Giemsa stain (#109204; Merck) for 30 min. Finally, the dye was removed by washing the cells with distilled water. Photographs of the cells were subsequently taken, and the colony area was quantified with ImageJ (v1.42q) software. The percentage of the area with colonies in the DPDS-treated group relative to that in the vehicle control group were analyzed.

### Assessment of viability via trypan blue exclusion staining

The breast cancer cell lines MDA-MB-231 and MCF-7 were seeded at a density of 3 × 10^4^ cells in a 12-well plate overnight. Afterward, the indicated concentrations of DPDS were administered to the cells for 24 h of incubation. Cell viability was determined via trypan blue exclusion staining (HiMedia, #TCL046, 0.4%, Mumbai, India) and further analyzed with SigmaPlot^TM^ v12 software (Systat Inc., Chicago, IL, USA). The concentration of DPDS (20 µM) used in the ROS scavenging assay for morphological observations was based on the IC_50_ of DPDS reported in our previous study 29. The cells were pretreated with N-acetylcysteine (NAC; #38520-57-9; Sigma‒Aldrich), a ROS scavenger, for 6 h before they were treated with 20 µM DPDS to determine the role of intracellular ROS in DPDS-induced cell death.

### Field-emission transmission electron microscopy (FE-TEM)

A total of 5 × 10^4^ cells were seeded in a 100-mm Petri dish overnight, after which the cells were treated with 20 μM DPDS for 24 h. Afterward, sample preparation was conducted as described by Cheng et al. 36. The cells were fixed in 4% PFA at room temperature for 24 h. The cells were then washed and fixed in 1% osmium tetroxide for 2 h, dried in graded acetone, infiltrated, and finally embedded in epoxy resin. A Leica microtome (Leica RM2165, Japan) was used to cut ultrathin 70-nm sections, and the changes in the morphology of the breast cancer cells and their mitochondria after DPDS treatment were observed via FE-TEM (HITACHI HT-7700, Tokyo, Japan) at an accelerating voltage of 80 kV.

### Western blotting

The cells were seeded at a density of 5 × 10^5^ in 100 mm petri dishes at 37 °C in an incubator with 5% CO_2_. Afterward, the cells were subjected to treatment with the indicated concentrations of DPDS for 24 or 48 h. Prior to DPDS treatment for 24 h, the cells were pretreated with NAC, an ROS scavenger to combat oxidative stress, for 6 h. Total protein was extracted from total breast cancer cells with RIPA lysis buffer. A BCA protein assay kit (Pierce, Rockford, IL, USA) was applied, and the absorbance at an OD of 595 nm was measured to confirm the protein concentration. The protein lysates were subsequently separated via sodium dodecyl sulfate-polyacrylamide gel electrophoresis (SDS-PAGE) and transferred onto polyvinylidene difluoride (PVDF) membranes (Pall, Ann Arbor, USA). The membranes were blocked with 5% (w/v) skim milk at room temperature and immunoblotted at 4 °C overnight with primary antibodies against Alix (# 634501; BioLegend), LC3B (#2775s; Cell Signaling Technology), BIP (#A11366; ABclonal), IRE-1α (#3294; Cell Signaling Technology), CHOP (#15204-1-AP; Proteintech), β-actin (#66009-1-1 g; Proteintech) and GAPDH (#MAB374; EMD Millipore Corp.), followed by incubation with horseradish peroxidase (HRP)-conjugated secondary antibodies. The immunoreactive bands were detected via enhanced chemiluminescence (ECL; #K-12045-D50; Advansta). Digital chemiluminescence images were captured and analyzed using an Amersham™ Imager 600 (Cytiva Corporation). The signal intensities of specific proteins were quantified with ImageJ (v1.42q; http://rsb.info.nih.gov/ij) analysis software.

### Immunofluorescence

Immunofluorescence staining was performed as previously described 37. Briefly, 2 × 10^4^ cells were seeded in a 24-well plate and cultured at 37 °C in a 5% CO_2_ incubator overnight. Afterward, the cells were treated with the indicated concentrations of DPDS and cultured for 24 or 48 h. The cells were fixed with 4% PFA, washed with 1× PBS, permeabilized with 0.5% Triton X-100, and blocked with 1% BSA. The cells were then incubated with primary antibodies at 4 °C for 1 h, washed with PBS, and incubated with the corresponding secondary antibodies for 1 h. After being washed with PBS, the cells were observed with an Olympus IX71 inverted microscope.

### Assessment of ROS levels via immunofluorescence and flow cytometry

Cells were seeded at a density of 5 × 10^4^ in a 6-well cell culture plate and incubated at 37 °C with 5% CO_2_. After 24 h, the cells in each experimental group were treated with different concentrations of DPDS (0, 10, 20, and 30 μM), and the cells were cultured for an additional 24 h. After the culture medium was removed, the cells were stained in the dark at 37 °C with the oxidation-sensitive fluorescent dye DHE (dihydroethidium; #D-23107; Invitrogen) and Hoechst 33342 dye (bisbenzimide H 33342 trihydrochloride; #B2261; Sigma‒Aldrich) for 15 min. After being washed with PBS, the cells were observed with an Olympus IX71 inverted fluorescence microscope (Tokyo, Japan) to detect immunofluorescence. For flow cytometry-based ROS detection, DHE-stained cells were analyzed on a Guava EasyCyte flow cytometer with a 488 nm bandpass blue excitation filter and a 590 nm (red) barrier filter.

### Statistical analysis

All the data presented in this study were obtained from at least three independent experiments and are expressed as the mean ± standard deviation. Statistical significance was determined using one-way analysis of variance (ANOVA) followed by Tukey's honestly significant difference (HSD) post hoc test for multiple comparisons. A *p* value < 0.05 was considered to indicate a statistically significant difference.

## Results

### DPDS inhibits the clonogenicity of breast cancer cells

**Figure 1:**
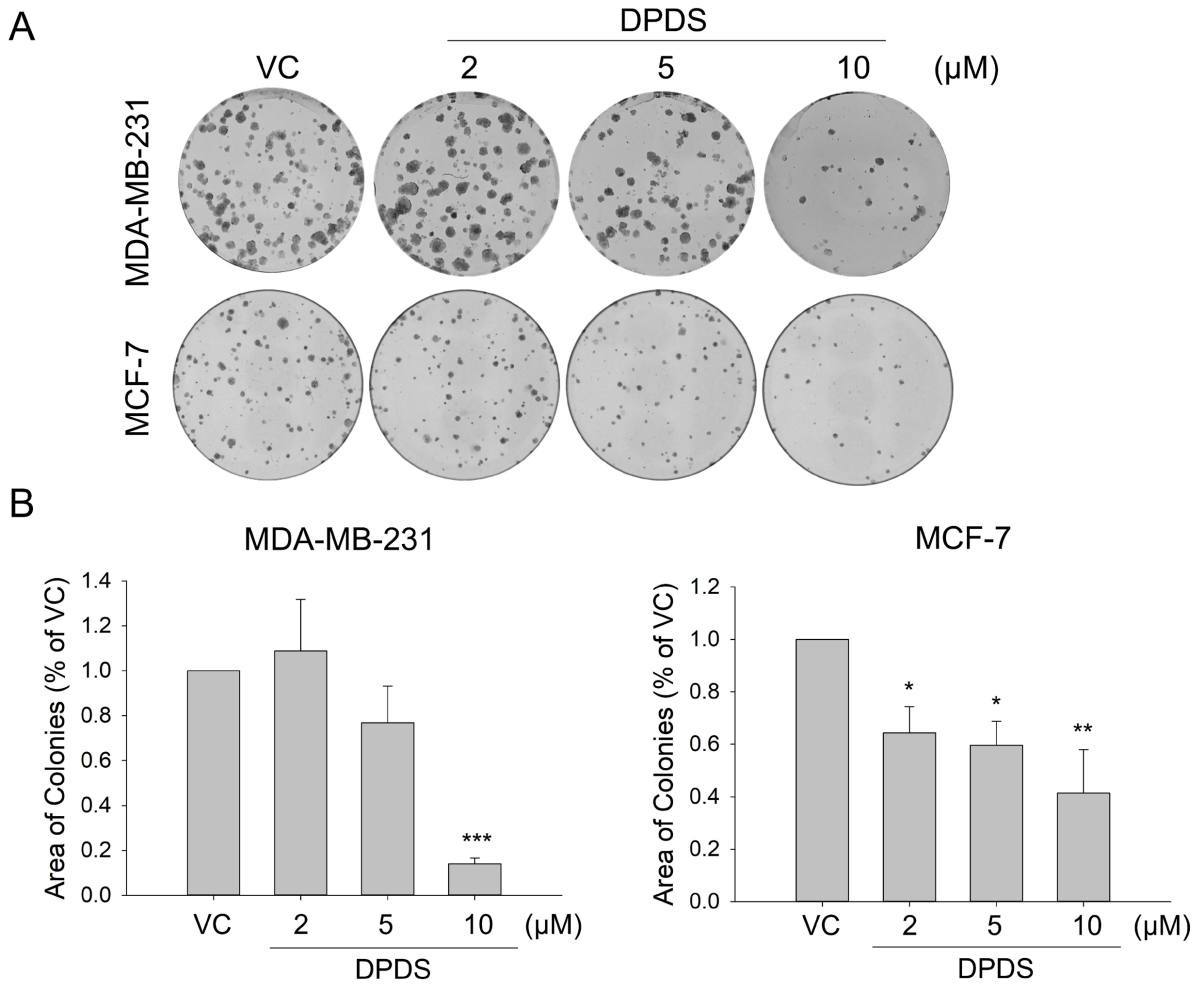
Assessment of breast cancer cell colony formation following DPDS treatment. The cells were treated with 2, 5, or 10 μM DPDS for 72 h, after which the medium was replaced. Ten days later, the colonies were stained with Giemsa dye, and the colony area was analyzed. (A) MDA-MB-231 and MCF-7 cells. (B) The quantitative results were statistically analyzed via one-way ANOVA. Vehicle control (DMSO was the solvent used to dissolve DPDS) vs. DPDS-treated groups. *p indicates a significant difference from the controls. *p < 0.05, **p < 0.01, and ***p < 0.001.

First, we assessed the impact of DPDS on two breast cancer cell lines, MDA-MB-231 and MCF-7. The results of the colony formation assay demonstrated that DPDS inhibited breast cell proliferation in a concentration-dependent manner (Figure 1A) and that this effect was statistically significant (Figure 1B).

### DPDS induces paraptosis in breast cancer cells

**Figure 2:**
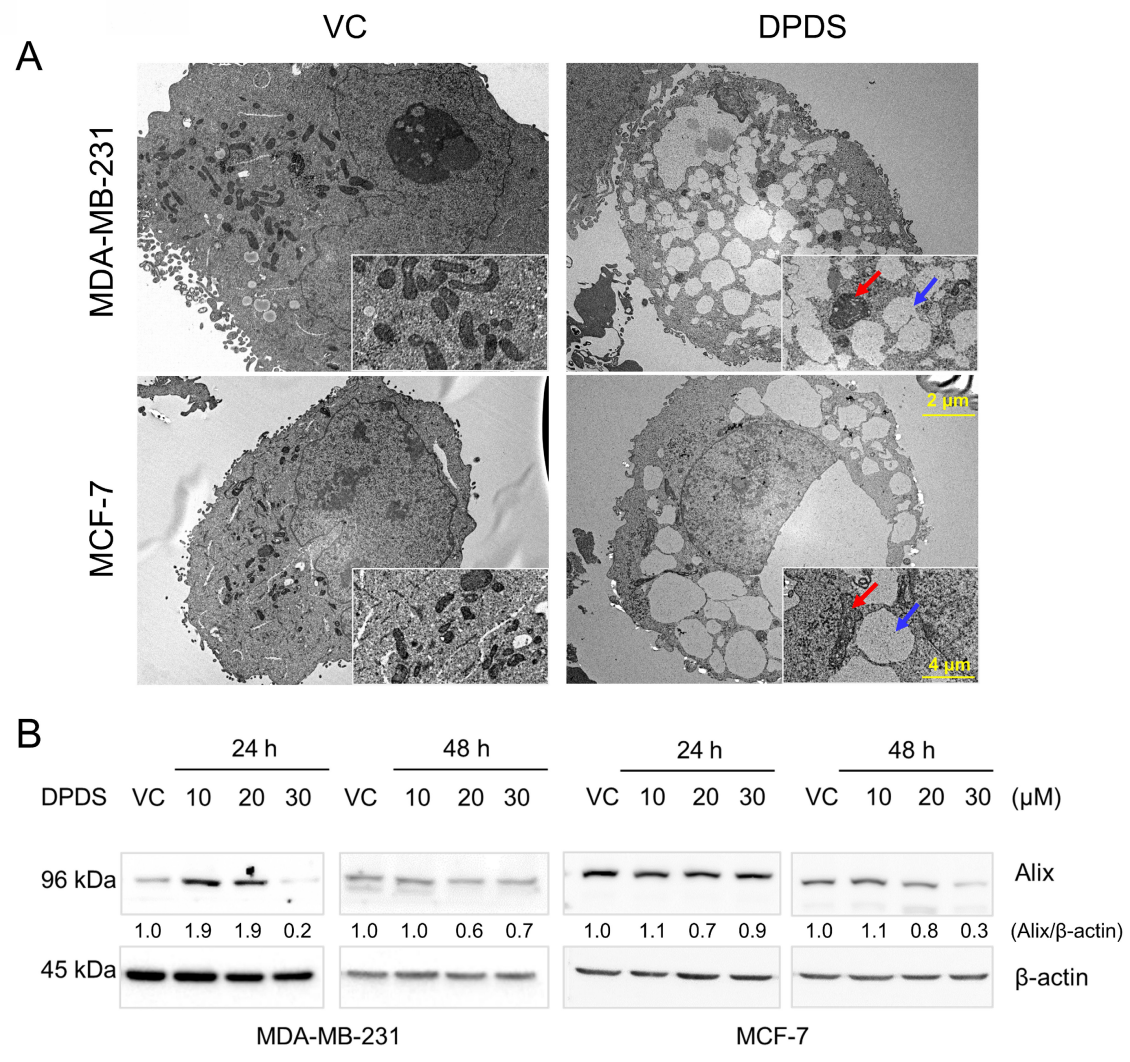
Characterization of paraptosis in breast cancer cells. (A) DPDS exposure induces a paraptotic phenotype in MDA-MB-231 and MCF-7 cells, as shown by FE-TEM. The blue arrows indicate the accumulation of cytoplasmic vacuoles (↑), and the red arrows indicate swollen mitochondria (↑) following DPDS administration for 24 h. Scale bars: 2 μm and 4 μm for the original and magnified images, respectively. (B) Western blot results showing that DPDS leads to a reduction in the expression of Alix, a hallmark of paraptosis. β-Actin was used as an internal control.

Paraptosis is characterized by cytoplasmic vacuolation, often accompanied by swelling of the ER and mitochondria 38. Studies have demonstrated that paraptosis involves the downregulation of AIP-1/Alix protein expression 12, 13, 32. The results demonstrated that exposure to DPDS caused vacuolation and mitochondrial alterations in MDA-MB-231 cells (Figure 2A). The expression level of Alix decreased with increasing concentrations of DPDS, indicating that DPDS indeed induces a form of cell death resembling paraptosis (Figure 2B).

### DPDS induces ER stress in breast cancer cells

**Figure 3:**
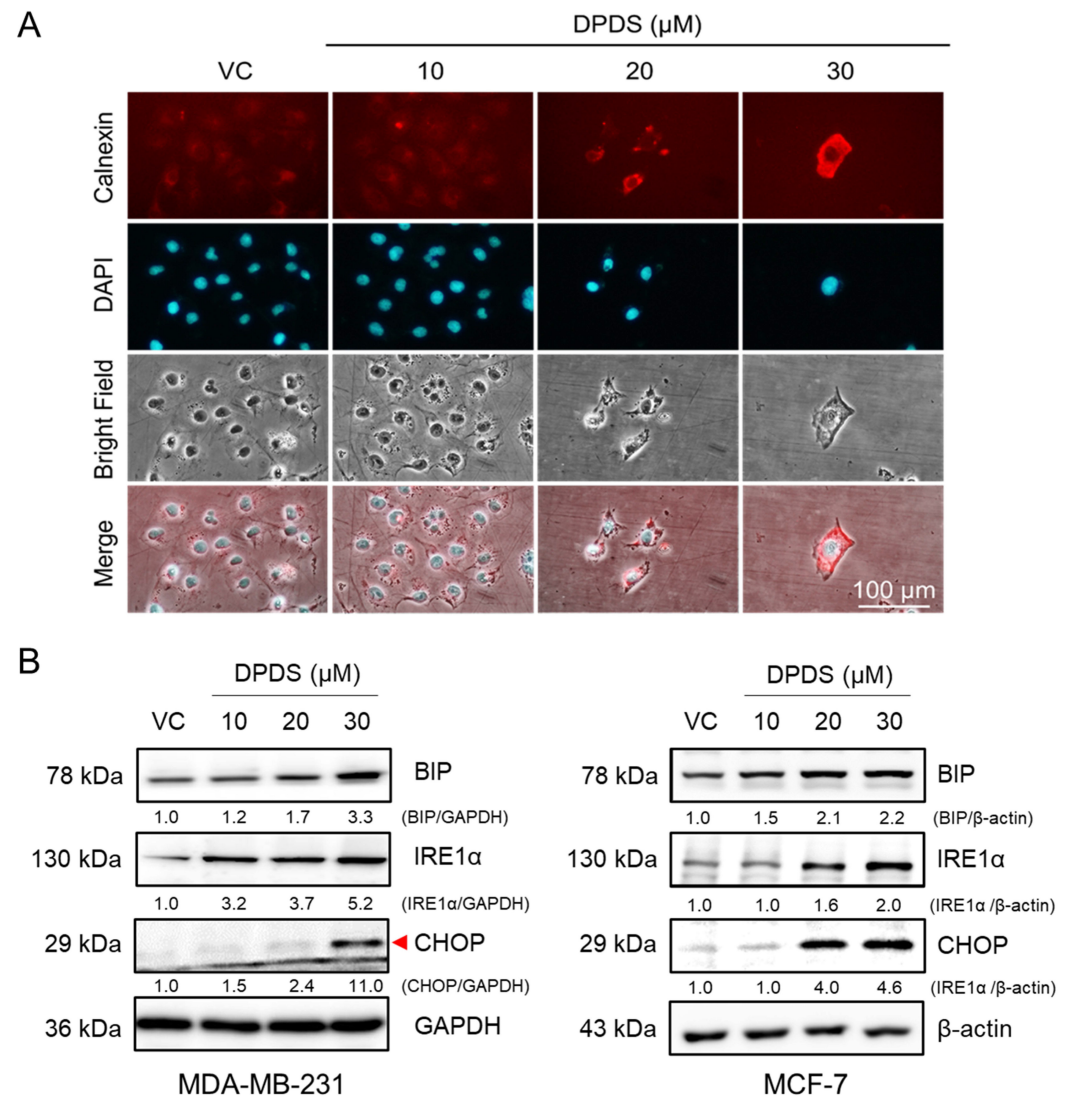
Effect of DPDS on ER stress-associated protein expression. (A) Immunofluorescence staining shows that DPDS increases the protein level of the ER marker calnexin in MDA-MB-231 cells. Magnification: 200 ×; scale bar: 100 μm. DAPI: blue fluorescence indicates the nuclei. (B) Western blotting revealed increased levels of ER stress-related proteins (BIP, IRE1α, and CHOP) in both MDA-MB-231 and MCF-7 cells. β-Actin was used as an internal control.

The ER is an organelle within the endomembrane system that plays a pivotal role in maintaining cellular homeostasis by facilitating processes such as protein translation, folding, and assembly. Under physiological conditions, the accumulation of misfolded proteins induces ER stress, subsequently activating the unfolded protein response (UPR), a signaling network that can promote either cell survival or apoptosis depending on the context 39. In addition, intracellular oxidative stress contributes to protein misfolding and subsequent ER stress 21, 22. Therefore, calnexin, a transmembrane ER protein involved in glycoprotein folding 40, 41, was used as a marker for immunofluorescence staining. The results demonstrated that exposure to DPDS increased calnexin levels in MDA-MB-231 cells (Figure 3A). Further analysis demonstrated that treatment with DPDS led to a dose-dependent increase in the expression of inositol-requiring enzyme 1 alpha (IRE-1α). Consistently, the levels of binding immunoglobulin protein (BIP), an ER-resident chaperone that senses misfolded proteins 42, and C/EBP homologous protein (CHOP), a proapoptotic transcription factor activated during ER stress 43, were also elevated in a concentration-dependent manner. Together, these findings indicate that DPDS induces ER stress in both the breast cancer cell lines examined (Figure 3B).

### ER stress partially alleviates DPDS-induced cell death and growth inhibition

**Figure 4:**
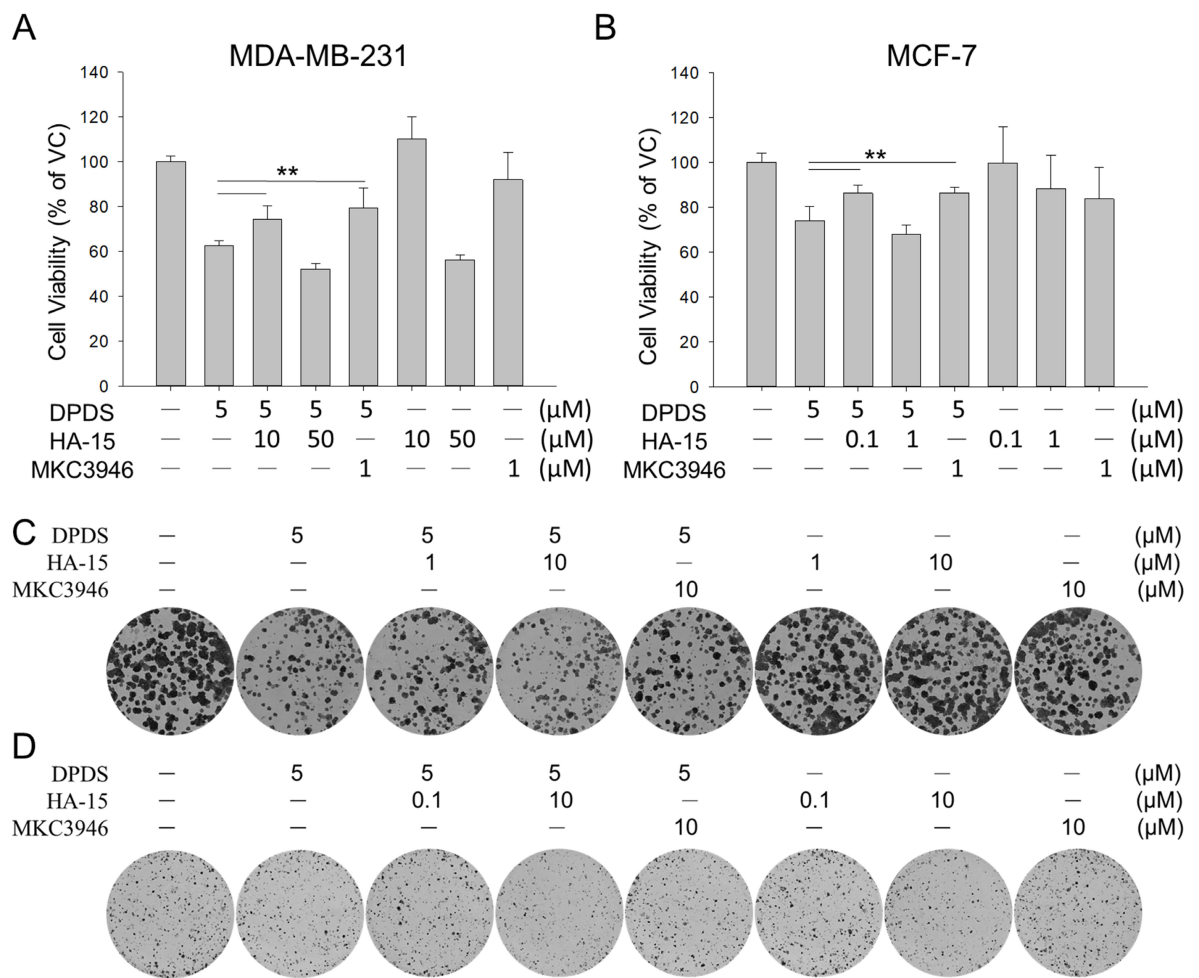
(A, B) Cell viability and (C, D) colony formation of MDA-MB-231 and MCF-7 cells treated with DPDS (5 μM) alone or in combination with MKC3946 (IRE1α inhibitor) or HA-15 (GRP78 inhibitor) for 24 h. For colony formation assays, cells were refreshed with drug-free medium post-treatment and incubated for ten days. The specific inhibition of IRE1α signaling by MKC3946 and the adaptive response triggered by noncytotoxic doses of HA-15 significantly attenuated DPDS-induced cell death and restored proliferative capacity. Vehicle control (DMSO was the solvent used to dissolve DPDS) vs. DPDS-treated groups. * indicates a significant difference from the controls. **p < 0.01.

To determine whether ER stress leads to breast cancer cell death upon treatment with DPDS, the two breast cancer cell lines were treated with DPDS and were treated with or without ER stress inhibitors (the GRP78 inhibitor HA15 and the IRE1α inhibitor MAC3946) for 24 h. Our data revealed that both ER stress inhibitors moderately rescued cell viability (Figure 4A-B) and colony formation ability (Figure 4C-D). These findings suggest that ER stress plays at least a partial role in DPDS-induced breast cancer cell death.

### DPDS induces paraptosis in breast cancer cells by inducing intracellular oxidative stress

**Figure 5:**
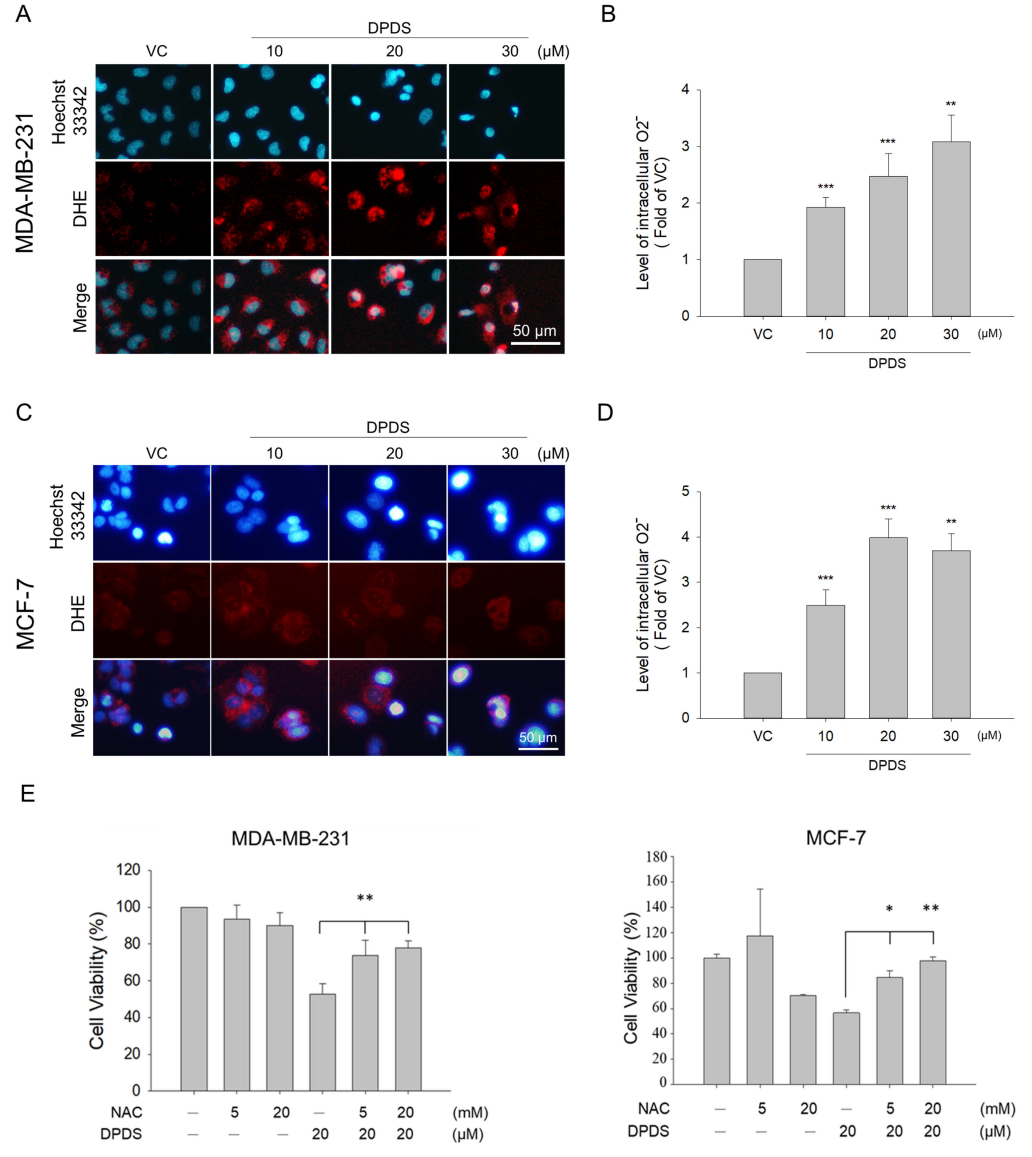
DPDS induces oxidative stress in breast cancer cells. The oxidative-sensitive fluorescent dye DHE was used to stain the cells to determine the intracellular ROS levels in (A) MDA-MB-231 cells and (C) MCF-7 cells; the cell nuclei were stained with Hoechst (blue). The ROS levels in (B) MDA-MB-231 cells and (D) MCF-7 cells were quantitatively analyzed via DHE staining and flow cytometry. Hoechst 33342 (blue) was used for nuclear staining. (E) NAC restored the viability of MDA-MB-231 and MCF-7 cells after treatment with DPDS for 24 h. The quantitative results were statistically analyzed via one-way ANOVA. **p < 0.01, DPDS alone vs*.* NAC pretreatment. Magnification: 200×; scale bar: 50 μm. * Indicates a significant difference from the controls. *p < 0.05, **p < 0.01, and ***p < 0.001.

Previous studies have shown that low levels of ROS can help cells maintain their normal physiological functions. However, excessive oxidative stress can cause cellular damage 19, 20. Many cancer cells exhibit higher levels of oxidative stress than normal cells do 44, 45. To investigate whether DPDS can increase oxidative stress in breast cancer cells, MDA-MB-231 and MCF-7 breast cancer cell lines were treated with different concentrations of DPDS (10, 20, and 30 μM) for 24 h, and the intracellular O_2_^-^ levels were determined via DHE staining. The results revealed that the intracellular ROS levels increased with increasing DPDS concentration (Figure 5 A-D). As shown in Figure 4E, when cells were pretreated with NAC, a ROS scavenger, to determine the role of oxidative stress induced by DPDS treatment, NAC significantly rescued cell viability, suggesting that DPDS-induced oxidative stress promotes breast cancer cell death (Figure 5E).

### DPDS induces ER stress in a ROS-dependent manner

**Figure 6:**
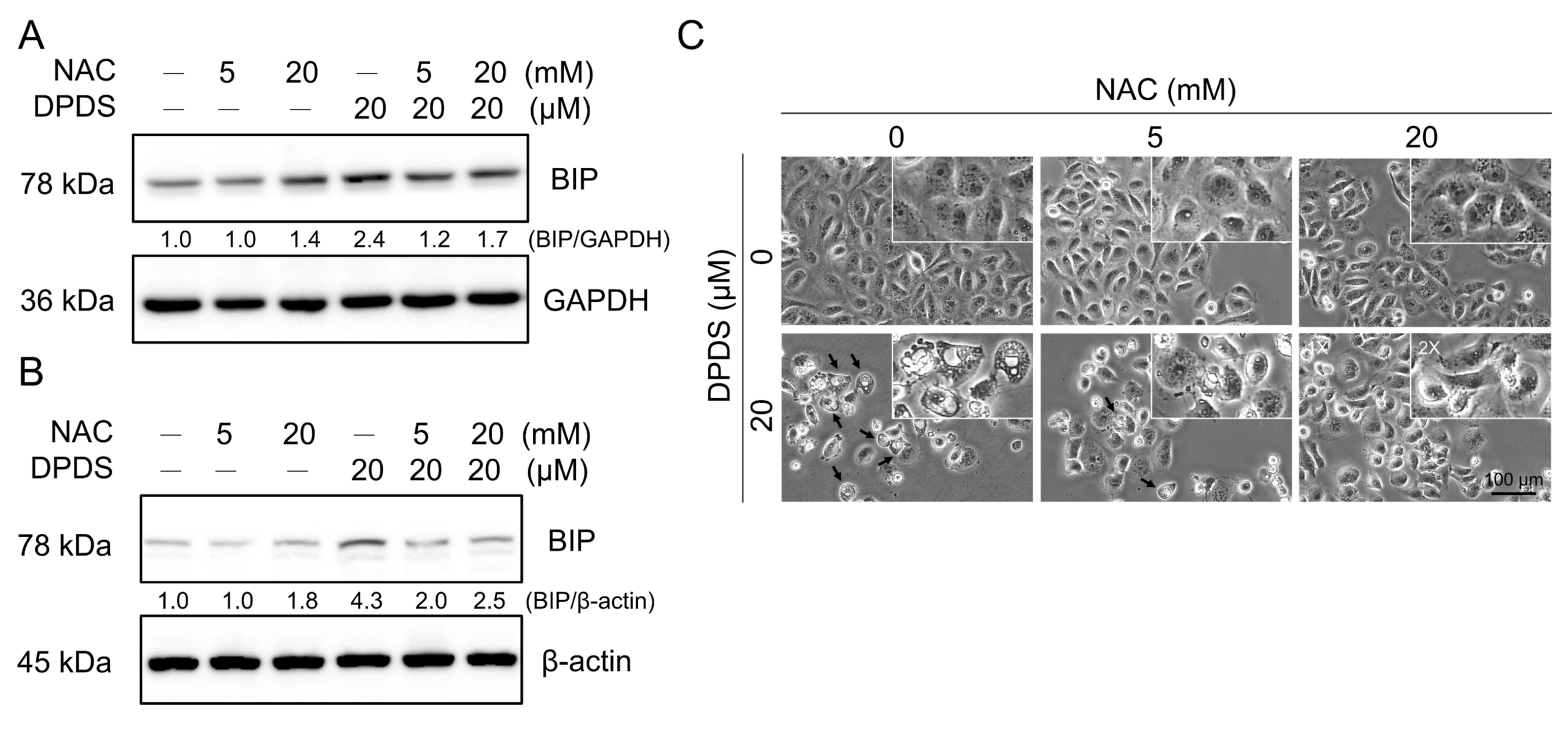
Role of ROS in DPDS-induced ER stress. NAC reduced DPDS-induced ER stress in (A) MDA-MB-231 and (B) MCF-7 cells. (C) MDA-MB-231 cells were treated with DPDS alone or in combination with NAC, a reactive oxygen species (ROS) scavenger. DPDS caused the accumulation of cytoplasmic vacuoles, a feature of paraptosis; however, NAC pretreatment reduced vacuole formation. Scale bar: 100 μm.

Our experimental results demonstrated that DPDS treatment induced ER stress (Figure 3). To investigate whether this induction was mediated by increased intracellular oxidative stress, we cotreated breast cancer cells with the ROS scavenger NAC, and the results revealed that NAC significantly attenuated DPDS-induced BIP/GRP78 expression (Figure 6A-B). Furthermore, ROS scavenging by NAC significantly mitigated DPDS-induced cytoplasmic vacuolization (Figure 6C) and restored the expression of the paraptosis marker Alix (Supplementary Figure 1). Taken together, these findings suggest that DPDS triggers ER stress and, subsequently, paraptosis through a ROS-dependent pathway.

## Discussion

DADS has been shown to suppress the growth and metastasis of triple-negative breast cancer (TNBC) cells 46, 47. However, the clinical utility of DADS is hindered by its high volatility, low bioavailability, and extremely short half-life under physiological conditions 48. In contrast, DPDS structure contains two phenyl groups connected by a disulfide bond, which provides superior chemical stability and greater potential for structural modification 49. Furthermore, both DADS and DPDS activate apoptotic pathways, while DPDS specifically promotes the cleavage of Bax p21 into its potent proapoptotic fragment p18 29. Specific Bax p21/p18 cleavage has been reported to significantly increase apoptotic cell death, which may be instrumental in overcoming apoptosis resistance in cancer cells 50, 51. In addition, several types of nonapoptotic programmed cell death, including ferroptosis, pyroptosis, and paraptosis, have been reported to restore chemosensitivity by bypassing dysregulated apoptosis 52, 53. Moreover, given that exosomal miRNAs play critical roles in tumor-immune interactions and the maintenance of the tumor microenvironment 54, it would be helpful for future studies to examine whether DPDS-induced paraptosis also influences exosome release, potentially enhancing the immune response against breast cancer cells. In our study, we demonstrated the potential of DPDS to exert antitumor effects through the induction of paraptosis, highlighting its therapeutic potential for treating breast cancer patients.

Paraptotic features in cells include cytoplasmic vacuolation and dilation of the ER and mitochondria 38. The initiation of paraptosis-like processes is associated with the downregulation of the endogenous apoptosis-inhibiting protein AIP-1/Alix 12, 13, 32. Given the heterogeneity of breast cancer tumors, we used two cell lines, namely, MDA-MB-231 and MCF-7, which exhibit distinct molecular characteristics, in our study. MDA-MB-231 cells do not express progesterone receptor (PR), estrogen receptor (ER), and human epidermal growth factor receptor 2 (HER-2); exhibit an aggressive phenotype; and are associated with poor prognosis 55. A previous study demonstrated that indirubin-3'-monoxime (I3M; a derivative of an active Chinese medicine component) triggered paraptosis in MDA-MB-231 (*p53*-mutant) cells in an ER stress-dependent manner, rather than inducing apoptotic cell death 56. In contrast, I3M can trigger apoptosis through p53-mediated ROS generation in HCT116 (*p53*-wt) cells 57. Nevertheless, Li et al*.* reported that ginsenoside Rh2 can initiate p53-dependent paraptosis in CRC cells, whereas vacuole formation is reduced after p53 knockout 58. These studies suggest that paraptosis induction might be independent of p53 status but dependent on the cellular context. Consistent with these findings, we found that DPDS-mediated paraptosis clearly occurred in both MDA-MB-231 and MCF-7 cells and was characterized by cytoplasmic vacuolation, mitochondrial dilation (Figure 2A), and the downregulation of Alix (Figure 2B), regardless of p53 status.

Furthermore, paraptosis is strongly correlated with ER stress. The accumulation of calnexin, an ER-associated chaperone, is frequently observed during paraptosis 40, 41, 59, 60. Similarly, our results revealed that exposure to DPDS increased calnexin expression in MDA-MB-231 cells (Figure 3A). The ER stress inhibitor partially protected against the DPDS-induced reduction in cell viability (Figure 4), suggesting that ER stress is involved in DPDS-induced cell death. Under normal physiological conditions, the accumulation of a small quantity of misfolded proteins can upregulate the expression of chaperone proteins, decrease protein translation, and arrest the cell cycle, allowing cells to repair or remove abnormally accumulated proteins. In contrast, a severe accumulation of misfolded proteins induces apoptosis 39. ER stress has been reported to not only induce apoptosis but to also trigger paraptotic cell death in breast cancer 60.

Several published reports have presented similar results. For example, a study revealed that combined treatment with auranofin and bortezomib synergistically inhibited canine mammary tumor cell lines through the induction of ER stress and paraptosis-like cell death 61. Moreover, glabridin, a prenylated isoflavonoid found in *G. glabra* L., has been reported to promote ER stress by upregulating the ER markers BIP, XBP1s, and CHOP in human breast cancer cells 62. Our results revealed that DPDS promoted ER stress in both breast cancer cell lines examined (Figure 3), which was accompanied by marked upregulation of BIP, IRE-1α, and CHOP expression (Figure 3B).

In this study, the involvement of ER stress in DPDS-induced cytotoxicity was validated using two distinct pharmacological approaches (Figure 4). First, the rescue effect of MKC-3946, a selective inhibitor of IRE1, suggests that the overactivation of IRE1 signaling is a primary driver of DPDS-mediated cell death. Second, we observed a protective effect of low-dose HA-15, which appears paradoxical given its role as a GRP78 inhibitor that typically induces stress. However, this observation aligns with the concept of ER hormesis, where sublethal modulation of the activity of GRP78 could activate adaptive UPR pathways without triggering the consequent cell death 63. Such preconditioning likely enhances the capacity of protein folding or degradative pathways such as autophagy, therefore attenuating the DPDS-induced proteotoxic burden. The fact that both pathway inhibition (MKC-3946) and adaptive priming (low-dose HA-15) significantly alleviated the cytotoxicity of DPDS provides evidence that maintaining ER homeostasis is critical for cell survival against DPDS-induced challenges. Furthermore, this biphasic response of HA-15 may highlight the complexity of GRP78 regulation and suggests that the intensity and duration of UPR activation determine the fate of cells under stress.

Excessive ROS production can disrupt redox homeostasis in the endoplasmic reticulum (ER), leading to the accumulation of misfolded proteins and the activation of ER stress 64, 65. The relationship between ROS and ER stress is bidirectional and complex and involves several mechanisms, with one amplifying another. Our study revealed the relationship between these two factors in the induction of paraptosis in breast cancer cell lines. Several published reports have presented similar results. For instance, camphene, a natural monoterpene, was found to induce ER stress by increasing ROS levels and upregulating CHOP, which led to the loss of mitochondrial membrane potential, and to increase caspase-3 activity, triggering the intrinsic apoptosis pathway, in MDA-MB-231 cells 66. Similarly, Ghosh and colleagues demonstrated that Withafarin, a natural steroidal lactone, triggered ROS-mediated swelling and mitochondrial fusion as well as ER dilation, leading to ER stress and paraptosis in MCF-7 and MDA-MB-231 cells 32.

Our results indicated that DPDS increases the O_2_^-^ content in breast cancer cells and induces intracellular oxidative stress (Figures 5A to 5D). NAC pretreatment significantly increased the survival of breast cancer cells treated with DPDS, which confirms the ROS-mediated cytotoxicity of DPDS (Figure 5E); reduced the total BIP expression level after DPDS treatment (Figures 6A and 6B); and effectively inhibited the formation of cytoplasmic vacuoles (Figure 6C). Our findings demonstrate that DPDS-induced ROS are the primary drivers of the morphological changes in and subsequent death of breast cancer cells, which are often associated with intense ER stress and autophagic processes 67, 68.

Therefore, our study further demonstrated that DPDS not only has the potential to induce apoptosis but can also induce nonapoptotic paraptosis through oxidative and ER stresses. Thus, it could be applied alone or in combination with current cancer drugs, especially for treating apoptosis-resistant cancers.

## Conclusion

DPDS effectively inhibited the growth of both MDA-MB-231 (TNBC) and MCF-7 (luminal) breast cancer cells, regardless of their gene status. Mechanistically, DPDS-induced ROS generation triggered ER stress, which was accompanied by alterations in the expression of several markers, including BIP, IRE-1α, and CHOP. Furthermore, ER stress facilitated the initiation of paraptosis in breast cancer cells, which was characterized by significant cytoplasmic vacuolation and the downregulation of Alix expression. In addition to apoptosis induction, our study revealed that DPDS initiated paraptosis, demonstrating its potential as a future treatment for breast cancer.

## Supplementary Material

Supplementary figure.

## Data Availability

All the data generated or analyzed during this study are included in this published article. Further inquiries can be directed to the corresponding authors.

## Abbreviations

BAG3: BAG cochaperone 3
BIP: binding immunoglobulin protein
CHOP: C/EBP homologous protein
CRC: colorectal cancer
DADS: diallyl disulfide
DHE: dihydroethidium
DPDS: diphenyl disulfide
ECL: enhanced chemiluminescence
ER: estrogen receptor
GSDME: gasdermin E
HER-2: human epidermal growth factor receptor 2
Hoechst 33342: bisbenzimide H 33342 trihydrochloride
HRP: horseradish peroxidase
IRE-1α: inositol-requiring enzyme 1 α
NAC: N-acetyl-l-cysteine
PFA: paraformaldehyde
PR: progesterone receptor
PVDF: polyvinylidene fluoride
ROS: reactive oxygen species
SRSF1: SR-rich splicing factor 1
TNBC: triple-negative breast cancer
UPR: unfolded protein response
XIAP: X-linked inhibitor of apoptosis
