# Supplementary material for: Diphenyl Disulfide Induces Nonapoptotic Paraptosis in Breast Cancer Cells via ROS-Mediated ER Stress
Source: Int J Med Sci. 2026 May 1;23(6):2096–107. doi: 10.7150/ijms.123945 (PMC13181374; doi:10.7150/ijms.123945)
Supplement: Supplementary file 1 — Supplementary figure. [file ijmsv23p2096s1.pdf]

Supplementary Figure

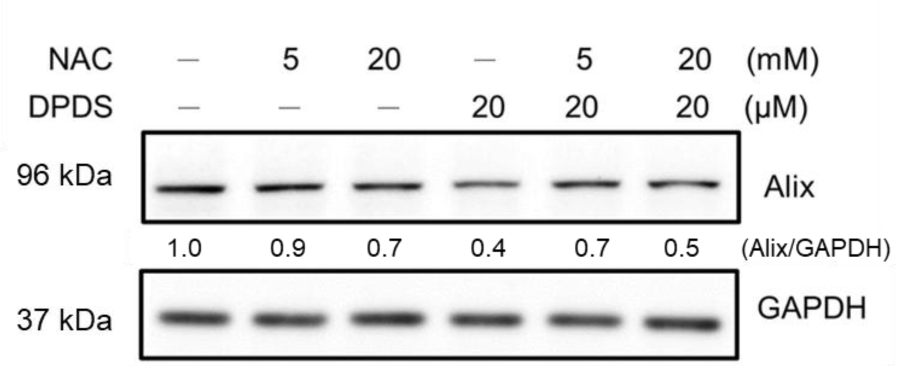

Supplementary Figure 1. In MCF-7 cells, treatment with DPDS significantly downregulated the expression levels of the paraptosis marker, Alix. However, pretreatment with NAC moderately upregulated Alix expression, suggesting that the DPDS-induced Alix downregulation could be ROS-dependent.
